# Nonthermal Atmospheric Plasma Modulates Palatal Wound Healing in Rats: A Morphometric, Histopathologic and Immunohistochemical Analysis

**DOI:** 10.3390/biomedicines14010089

**Published:** 2026-01-01

**Authors:** Basak Kusakci Seker, Hakan Ozdemir, Suna Karadeniz Saygili

**Affiliations:** 1 Dentarma Private Oral and Dental Health Clinic, 07000 Antalya, Türkiye; drbasakseker1@gmail.com; 2 Department of Periodontology, Faculty of Dentistry, Eskisehir Osmangazi University, 26000 Eskisehir, Türkiye; hozdemir52@hotmail.com; 3 Department of Histology and Embryology, Faculty of Medicine, Kutahya Health Science University, 43000 Kutahya, Türkiye

**Keywords:** fibroblast, growth factors, non-thermal atmospheric plasma, plasma medicine, wound healing, plasma-induced regeneration, oral mucosa, angiogenesis

## Abstract

**Background/Objectives:** Non-thermal atmospheric plasma (NTAP) has recently gained attention as a promising tool for tissue regeneration due to its ability to modulate cellular signaling and enhance wound repair. However, its effects on oral mucosal healing and associated molecular pathways remain insufficiently characterized. This study aimed to investigate the histological and immunohistochemical effects of NTAP on palatal wound healing in rats and to evaluate key biomarkers involved in angiogenesis, proliferation, and extracellular matrix remodeling. **Methods:** Sixty rats were randomly assigned to three groups: Saline Control Group (SCG), Chlorhexidine Gluconate Group (CHG), and NTAP-Treated Group (NTAPG). Standardized full-thickness excisional wounds were created in the central palatal mucosa. Animals were sacrificed on postoperative days 7, 14, and 21. Histological assessments included vascularization, inflammatory cell infiltration, collagen fiber organization, and epithelial gap measurements. Immunohistochemical analyses were performed using antibodies targeting VEGF-A, TGF-β, FGF-2, CD34, α-SMA, and Ki67 to evaluate angiogenesis, fibroblast activity, and cellular proliferation. **Results:** NTAP treatment significantly elevated TGF-β levels at all time points and increased α-SMA-positive cell counts on days 7 and 14. FGF-2 expression was the highest in NTAPG, while VEGF-A and CD34 levels were significantly elevated, indicating robust angiogenic activity. NTAP also reduced inflammatory cell infiltration relative to the other groups. NTAPG exhibited enhanced fibroblast proliferation, increased collagen deposition, improved vascularization, and accelerated re-epithelialization compared with SCG and CHG. **Conclusions:** NTAP significantly promoted palatal wound healing by enhancing proliferative activity, stimulating growth factor expression, and accelerating tissue repair. These findings suggest that NTAP may serve as an effective therapeutic approach for improving oral mucosal wound healing.

## 1. Introduction

There has been great interest in the control of tissue repair after intraoral surgical procedures in periodontology. It is now clear that oral wound healing is scar-less and takes place more quickly than healing on dermal tissue. However, in the oral cavity, masticatory trauma on the wound site and bacterial infection may negatively affect the normal healing process. Oral soft tissues are inevitably damaged during periodontal surgery, dental implant surgery, or autogenous gingival grafting. Palatal masticatory mucosa has been extensively used as a tissue donor area for epithelial soft tissue grafts for treatment of gingival recession and increasing the keratinized mucosa. The major complication of these surgical techniques is the formation of a secondary wound, which may show a prolonged healing process; in addition, postoperative discomfort, oedema, and pain associated with an open wound site can sometimes occur [1]. Recent studies have reported donor-site infection rates of approximately 2–8% following palatal soft tissue harvesting, suggesting that prolonged wound healing may further increase the risk of postoperative infection [2]. Therefore, it is necessary to ensure that the wounds in the oral cavity are closed as soon as possible. Although a variety of therapeutic strategies have been adopted in clinical practice for oral wound healing, there is a need for alternative and effective therapies due to long-term applications and side effects [3]. For all these reasons, physical therapy to accelerate healing by applying physical energy to wound areas has recently attracted attention. Furthermore, non-thermal atmospheric plasma (NTAP) has been considered as a potential tool for wound management due to its beneficial effects on wound healing [4]. Plasma is defined as the fourth state of matter formed by adding energy to a gaseous substance. There are a variety of plasma devices that use different inert gases (such as helium or argon) to produce NTAP. The most important feature of NTAP that causes several physical and chemical changes in both inorganic and organic structures is the production of ultraviolet, free radicals, ozone and reactive oxygen and nitrogen species with the application of NTAP. Through these products, NTAP can perform biostimulation, biomodification, and antimicrobial activities; therefore, they can be used for various purposes in biomedical fields, medicine, and dentistry [5,6].

During the wound-healing process, a wide variety of growth factors (e.g., vascular endothelial growth factor (VEGF), fibroblast growth factor 2 (FGF2) and transforming growth factor ß (TGF-ß)) are released into the extracellular matrix of the local tissues following injury due to surgical procedures. This group of early growth factors are essential for wound healing and stimulating granulation tissue formation, angiogenesis, migration and proliferation/activation and re-epithelization [7].

NTAP can increase the expressions of various growth factors and cytokines in human fibroblasts and keratinocytes with the reactive oxygen and nitrogen species (ROS/RNS), which make it biologically active. The beneficial effects of NTAP on wound healing have been assessed in several in vivo studies and case reports [8,9]. In these studies, the results showed significant improvement in the healing of pressure ulcers using plasma, and faster size reduction of wounds after three weeks compared to the group without plasma therapy [10].

Despite the advances in plasma research, there still remain many questions regarding the effects of cold plasma on cellular physiology in gingival tissues. When the literature was reviewed, a limited number of immunohistochemical studies were found that examine the effects of plasma on cellular mechanisms in wound healing and also, most of these are cell culture studies [11,12]. On the other hand, in studies on animals, the relevant factors were investigated piece by piece, not as a whole. For example, in the Choi et al.’s study, immunohistochemistry assays were performed to determine the level of various growth factors, including TGF-α, TGF-β, VEGF, GM-CSF, and EGF, in mice skin tissue. Also, in another study, Arndt et al. performed immunohistological analyses which were directed against CD68 anti-alpha-smooth muscle actin (alpha-SMA) and Ly6G [11]. However, in order to clearly see the results of plasma applications, it is very important to determine molecular changes and related mechanisms. The aim of this histomorphometric and immunohistochemical study was to evaluate the effects of NTAP on the healing of excisional wounds in palatal mucosa of rats, and to analyze the expressions of some factors related to wound healing.

## 2. Materials and Methods

### 2.1. Experimental Animals

A total of sixty, 12-week-old male Wistar albino rats (weighing between 200 and 220 g) obtained from the Medical and Surgical Experimental Animals Application and Research Centre of Eskisehir Osmangazi University were used in the experiment. The experimental protocol and design were performed in accordance with guidelines laid down by the National Institutes of Health in the United States regarding the care and use of animals for experimental procedures or with the European Communities Council Directive (2010/63/EU) and approved by the Animal Research Ethics Committee of Eskisehir Osmangazi University (Protocol No. 669-1). The study protocol and the manuscript were created according to the “NC3Rs ARRIVE Guidelines, Animal Research: Reporting of In Vivo Experiments.”

All animals were maintained in a 12 h light/12 h dark cycle, at a temperature of 22 ± 1 °C and fed a standard diet of pellets and allowed free access to water. The rats had been acclimatized to their living environment for 1 week before the experiment to reduce the stress level. Six animals were sacrificed immediately and provided the baseline group at time 0. Then, the animals were randomly divided into three groups as follows: Saline (0.9%) Control group (SCG) (*n* = 18), Chlorhexidine gluconate (0.2%) (CHG) group (*n* = 18), and Non-thermal atmospheric plasma group (NTAPG) (*n* = 18). Additionally, the groups were further divided into three subgroups (containing 6 rats in each) for sacrifice on the 7th, 14th, and 21st days of wound healing.

### 2.2. Surgical and Treatment Protocol

The animals were anaesthetized intraperitoneally with a mixture of 10% ketamine hydrochloride (Ketalar; 40 mg/kg, Eczacibasi Pharmaceutical Industry, Istanbul, Türkiye) and 2% xylazine hydrochloride (Rompun; 10 mg/kg, Bayer Animal Health GmbH, Leverkusen, Germany). After anesthesia, full-thickness 3 mm-diameter standardized excisional wounds were created in the center of the palatal mucosa using a 3 mm-diameter biopsy punch (BP-30F, Kai Medical, Gifu, Japan). Mucoperiosteal tissues were removed by sharp dissection to expose the underlying bone area for secondary healing, and hemostasis was achieved by compression after wound preparation [13].

Following 2 h after the wounding procedure, kINPen 11 plasma jet (Leibniz Institute for Plasma Science and Technology, Greifswald, Germany) was applied to the wounds in the NTAPG. The plasma jet comprises a base station including the control unit and the power supply, together with a hand piece containing the electrical discharge system and the gas flow. The discharge system is supplied with argon plasma, with the pulses being generated at a frequency of 21 kHz with an applied voltage of 5 kV. The plasma stream is approximately 10 mm long and has a plasma–tissue interaction zone of about 1.5 mm in diameter. Argon gas is used as the carrier gas at a flow rate of 5 L/min at 2.5 Bar. The distance between nozzle and wound was approximately 5 mm. The wounds were treated for 60 s with the plasma jet moving in three different directions (horizontal, vertical, and diagonal) over the wound surface and the application was performed at 2-day intervals following the wounding for a total of three periods. Electro-optical diagnostics from device calibration were employed to characterize the discharge behavior of the treatment. The kINPen^®^ system exhibited steady pulsed waveforms for both applied voltage and discharge current. The optical emission spectroscopy (OES) of the argon plume revealed characteristic Ar I lines (690–850 nm) and low-intensity signals of reactive oxygen and nitrogen species (RONS), consistent with typical argon non-thermal atmospheric pressure (NTAP) emission profiles [14]. Also a similar NTAP application protocol had been previously established and validated by our research group in an experimental periodontitis model [15].

**Figure 1:**
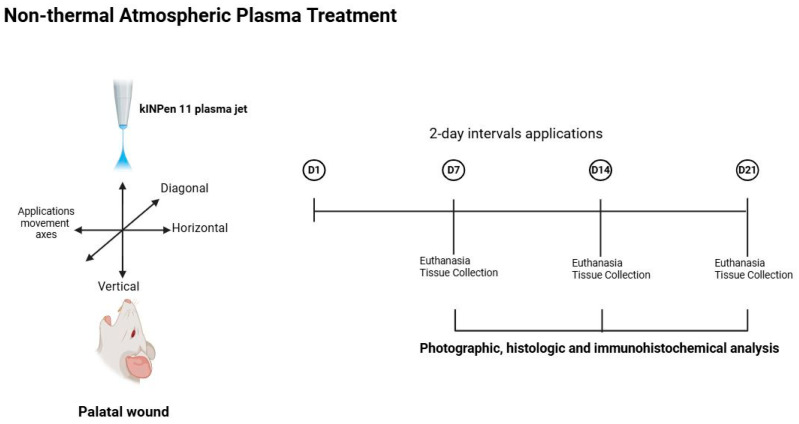
A schematic representation of the non-thermal atmospheric plasma (NTAP) treatment protocol. The kINPen^®^11 plasma jet was applied to the palatal wound surface using horizontal, vertical, and diagonal movement axes for 60 s. NTAP applications were performed at 2-day intervals following wounding. Tissue samples were collected on days 7, 14, and 21 for photographic, histologic, and immunohistochemical analyses. Created in BioRender. Saygili, S. (2025) https://BioRender.com/uk31nd0 (Accessed on 19 December 2025).

The CHG received a daily application of the 0.2% of chlorhexidine (Klorhex^®^, Drogsan Pharmaceuticals, Ankara, Türkiye), and SCG received a saline application daily. At 24 h postoperatively, mild anesthesia (i.m. injection containing 0.1 mL of 10% ketamine hydrochloride and 0.1 mL of 20% xylazine hydrochloride) was used to apply agents on the wounds every day as one dose per day until scarification was performed for each group. Without touching the wound, 1 mL of the tested agent was delivered directly to the wound using a syringe with a blunt cannula [16]. After 2 h of agent application, animals were fed a standard diet of pellets and water ad libitum. Six animals from each group were sacrificed by cervical dislocation after previous sedation with ketamine and xylazine at 7, 14, and 21 days, postoperatively. The maxillae were dissected out, and the samples were photographically assessed and compared with the histological and immunohistochemical findings (Figure 1).

### 2.3. Photographic Analysis

The images of the palatal specimens in all groups were captured by a digital camera which attached to a light microscope (DM 4000 B, Leica, Wetzlar, Germany, DC 500 camera, Leica, Wetzlar, Germany) with an original magnification of ×40 and with opening, intensity flash, exposure time, and distance all standardized. A scientific ruler was also placed within the photographic area to confirm accuracy. The digital photographs were transferred to a computer and then, wound margins were marked and the wound surface area was calculated in mm^2^ for each animal using the image analysis software Leica QWin Plus V 3.3.1 (Leica Microsystems GmbH, Wetzlar, Germany) where the ruler in the photograph was used as a scale reference. Only an experienced calibrated examiner (H.O.), who was blind to the samples, carried out the measurements. The researcher made two different measurements in 15 independent samples for to avoid calibration errors. The measurements of this study were carried out by the researcher when there was a r-value of 0.99 in the statistical evaluation of these measurements, and there was a 99% consistency between these measurements.

### 2.4. Histologic and Immunohistochemical Analysis

The specimens were fixed immediately in 10% formalin for at least 48 h and then decalcified in 10% ethylenediaminetetraacetic acid for 6 weeks. Samples were cleared in xylene and embedded in paraffin. Finally, the specimens were divided into two sections: one section was stained with hematoxylin–eosin (H&E) staining and the other sections with immunohistochemical staining. For each sample, 5 sections, each 5 μm thick, were cut in the transversal plane serially by using a microtome, then underwent deparaffinization and were rehydrated. The sections were stained with H&E for evaluation by light microscopy (Zeiss Axiovision, Oberkochen, Germany).

Histologically, the vascularization and inflammation rate were evaluated with those H&E sections and scored according to Kirchner et al. [17] as follows: 0 = absent; 1 = mild; 2 = moderate; and 3 = marked. The density of the collagen fibers in the specimens was determined at 4 degrees with the following scale: 1 = few collagen fibers; 2 = few and partially spread collagen fibers; 3 = few and fully spread collagen fibers; and 4 = dense collagen fibers [18]. In addition, morphometric analysis of wound surface areas and the distances between the epithelial margins in each section were measured with Zen software version 4.8.2 (Zeiss, Oberkochen, Germany) for investigating the rate of epithelialization of the palatal wound.

The tissue sections were prepared for immunohistochemical analysis according to these procedures: Firstly, tissues were deparaffinized, rehydrated through graded alcohol and rinsed in distilled water. Next, tissue sections were incubated with antigen retrieval solution (ab93684, Abcam, Cambridge, UK) for 15 min at 37 °C and treated with hydrogen peroxide blocking reagent (ab64218, Abcam, Cambridge, UK) for 15 min to diminish non-specific staining. After rinsing three times with phosphate-buffered saline (PBS) (ab64247, Abcam, Cambridge, UK), the slides were incubated with primary antibodies at 4 °C overnight, as follows: VEGF-A in a 1:200 dilution (ab1316, Abcam, Cambridge, UK); FGF2 in a 1:200 dilution (ab92337, Abcam, Cambridge, UK); TGF-β1 in a 1:200 dilution (ab190503, Abcam, Cambridge, UK); Cluster of differentiation 34 (CD34) in a 1:100 dilution (ab185732, Abcam, Cambridge, UK); α smooth muscle actin (α-SMA) in a 1:200 dilution (ab5694, Abcam, Cambridge, UK); and Ki67 in a 1:200 dilution (ab15580, Abcam, Cambridge, UK). After rinsing with PBS, the sections were incubated with secondary antibody HRP/DAB IHC Detection Kit (ab236466, Abcam, Cambridge, UK). To visualize the protein, the slides were incubated with the DAB substrate provided in the HRP/DAB IHC Detection Kit. Then the samples were counterstained with Mayer’s hematoxylin (Dako, Glostrup, Denmark) for analysis using a microscope. The immunoreactive cells were evaluated by a blinded and trained histologist (S.S.). Quantification of immunohistochemical staining intensities were performed using Fiji freeware (Image version 2.1.0, National Institutes of Health, Bethesda, MD, USA) as described by Patera et al. [19]. The average gray value was calculated from the second images obtained after color deconvulsion on five different microphotographs belonging to all groups. Optical Density (OD) was calculated with using the formula OD = log (maximum gray density/average gray density). For immunohistochemical quantification, marker expression was evaluated within the ulcer region when present. In specimens showing complete re-epithelialization, measurements were performed in the corresponding wound area previously identified in H&E-stained sections, ensuring consistency across groups and time points.

### 2.5. Statistical Analysis

MedCalc Software for Windows (Version 17.5) program (Broekstraat, Mariakerke, Belgium) was used for all statistical analyses. To define the normality, the Shapiro–Wilk statistical test was used. For normally distributed data, ANOVA, followed by post hoc (Scheffe) tests, was performed to determine the presence of any significant difference between groups. For non-normally distributed data, the Kruskall–Wallis and Dunn tests were used. *p* values less than 0.05 were considered to be statistically significant.

## 3. Results

**Figure 2:**
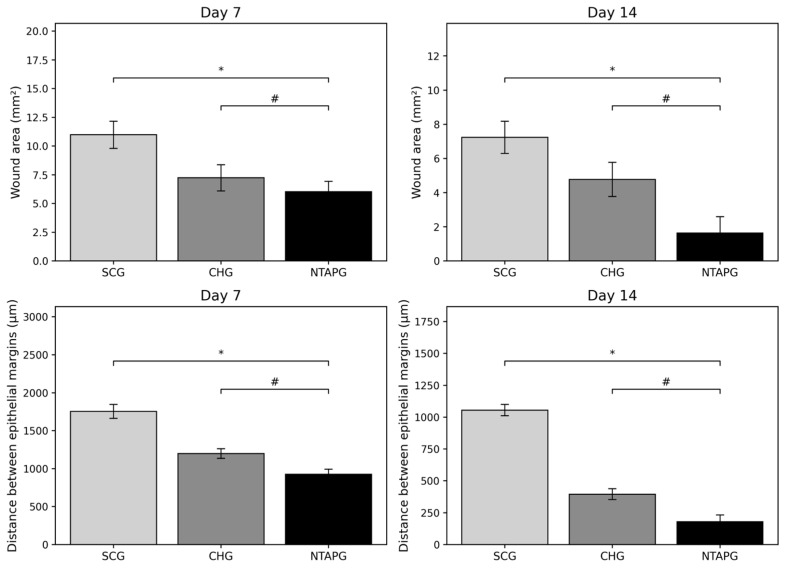
Wound surface areas and the distance between the epithelial margins in all groups at each time points after wounding. Data are shown as mean ± SD. *p* < 0.05 vs. SCG (*); *p* < 0.05 vs. CHG (#).

**Figure 3:**
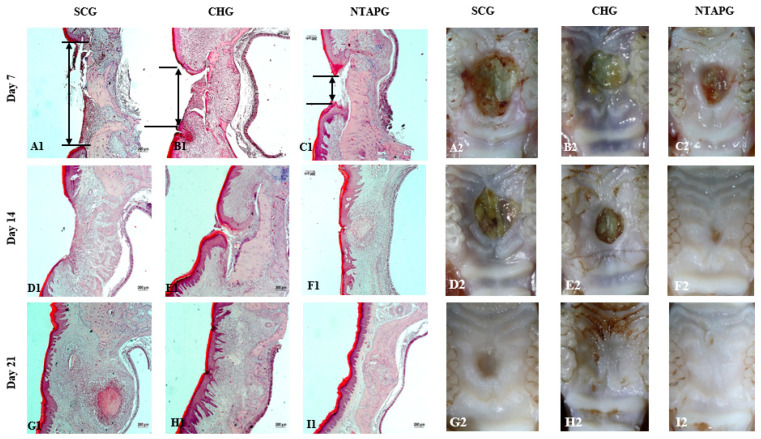
Representative images of H&E in the wounds of SCG, CHG, and NTAPG on day 7, 14, and 21 post-wounding (**A1**–**I1**). The double-headed black arrows indicate the distance between the epithelial margins. Seven and fourteen days after injury, the wound width in the NTAPG was significantly smaller than both groups (*p* ˂ 0.05) (**C1**,**F1**). Epithelialization was almost completed in all groups on day 21 (**G1**–**I1**). The representative light-microscopic photographs of the wound areas on 7th, 14th, and 21st days post-wounding (**A2**–**I2**). The most significant difference in wound surface areas among the groups was seen on day 14 (**D2**–**F2**); the scale bar was 200 μm.

The photographic images of wounds in all groups taken on days 7, 14, and 21 post-wounding are given in Figure 2. The wound surface area decreased over time in both groups. Seven days after injury, all groups showed a reduction in the wound area, and it was significantly lower in the NTAPG compared to the SCG (*p* ˂ 0.001). The most significant difference in wound surface areas among the groups was seen on day 14 post-wounding. In the NTAPG (1.64 ± 0.95 mm^2^), the remaining lower wound surface area was smaller compared to the chlorhexidine (4.78 ± 1.0 mm^2^) and control (7.24 ± 0.94 mm^2^) groups (*p* ˂ 0.001). According to photographic evaluation, the fastest wound healing was found in the NTAPG (Figure 2 and Figure 3; Table S1).

The degree of re-epithelialization was determined by measuring the distance between the epithelial margins. The distance between the epithelial margins decreased with time in all experimental and control groups. Seven and fourteen days after injury, the wound width in the NTAPG (926.66 ± 66.99 μm and 178.33 ± 54.36 μm) was significantly smaller than that of the CHG (1199.50 ± 63.98 μm and 395.50 ± 43.09 μm) and SCG (1753.50 ± 91.37 μm and 1054.83 ± 44.07 μm) (*p* ˂ 0.001). Epithelialization was completed in all groups on day 21 post-wounding (Figure 2 and Figure 3; Table S1).

**Figure 4:**
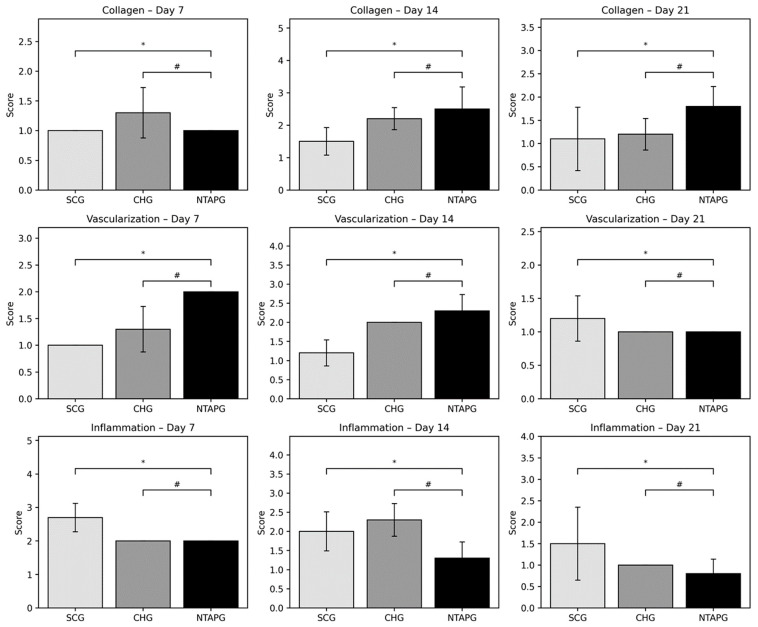
Collagen, vascularization, and inflammation scores in H&E sections of all groups at each time points after wounding. Data are presented as mean ± SD. *p* < 0.05 vs. SCG (*); *p* < 0.05 vs. CHG (#).

Epithelialization, inflammation, vascularization, and collagen deposition were analyzed from histological sections. When inflammation findings were compared on the 7th, 14th, and 21st day after injury, it was seen that the scores were significantly higher in the SCG compared to the NTAPG (*p* = 0.0012, *p* = 0.043 and *p* = 0.015). The least inflammation scores were observed in the NTAPG. Both groups had a remarkable decrease in inflammation with time. There was an increase in angiogenesis in the group treated with NTAP compared to both the SCG and CHG on days 7 and 14 after injury, but this difference is significant only in comparison with the SCG on day 7 (*p* = 0.002, *p* = 0.001). In both groups, a reduction in blood vessels was observed on day 21 compared with days 7 and 14 post-wounding. The density of collagen fibers was seen to be almost equal in all groups on day 7 after wounding, but in the SCG there was a more irregular collagen deposition. Fourteen days after injury, an increase in collagen fiber density was noted in both groups. Furthermore, the highest collagen deposition score was in the NTAPG, but the differences were not significant (*p* = 0.052). On the 21st day post wounding, there was a decreased fiber accumulation in all groups due to tissue organization. In addition, collagen accumulation was significantly higher in the NTAPG compared to the SCG (*p* = 0.036) (Figure 4; Table S2).

**Figure 5:**
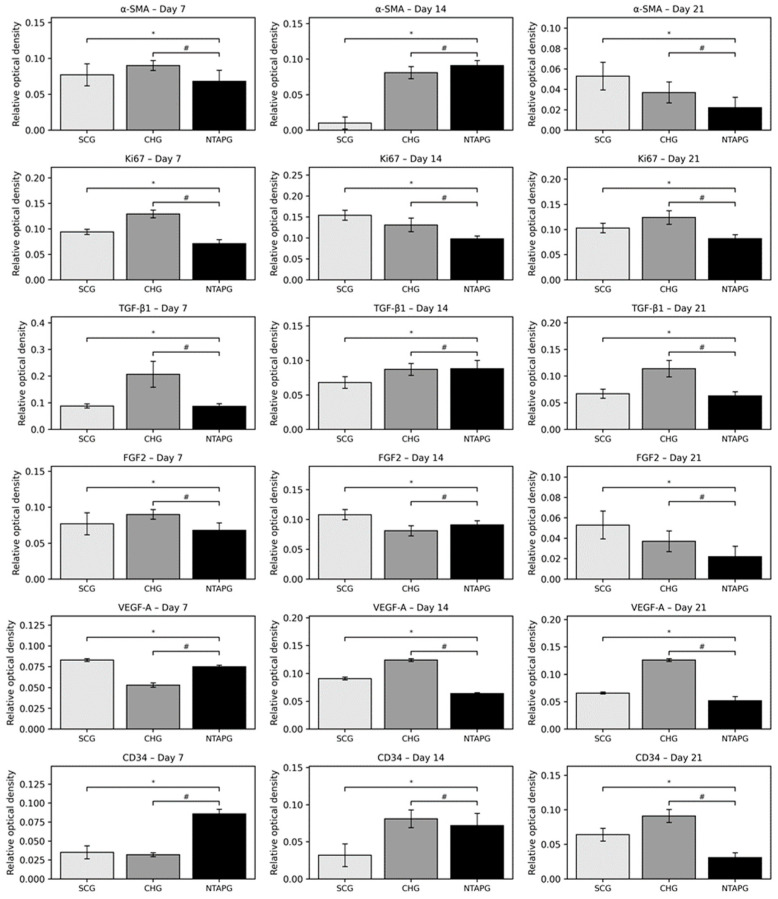
Relative optical density of α-SMA, Ki67, TGF-β1, FGF2, VEGF-A, and CD34 immunostaining at 7, 14, and 21 days post-wounding in all groups. Data are presented as mean ± SD. *p* < 0.05 vs. SCG (*); *p* < 0.05 vs. CHG (#).

**Figure 6:**
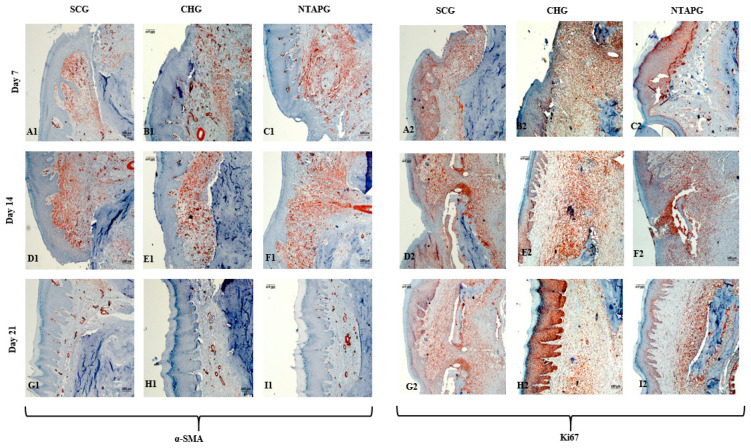
Immunohistochemical staining of α-SMA and Ki-67 in the SCG, CHG and NTAPG at post-wounding days 7, 14, and 21. Panels A1–C1, D1–F1, and G1–I1 show α-SMA staining in SCG, CHG, and NTAPG on days 7, 14, and 21, respectively. Panels A2–C2, D2–F2, and G2–I2 show Ki-67 staining in the same groups and time points. α-SMA-positive cells increased on day 14, with a significantly higher number observed in the NTAPG compared to the other groups (*p* < 0.05). Ki-67 expression increased on days 7 and 14 in all groups and decreased by day 21; although higher in the NTAPG, the differences were not statistically significant (*p* > 0.05). The magnification was ×100 in all the panels; the scale bar was 100 μm.

The paraffin tissue sections were analyzed by immunohistochemistry and the results were presented. On the 7th day after injury, α-SMA positive cells were present in both groups, especially in the submucosal area. The increase of α-SMA-positive cells was seen on day 14 and a significantly higher number of α-SMA-positive cells was present in the NTAPG on day 7 and 14 (*p* = 0.046 and *p* = 0.013). On the 21st day after injury, α-SMA positive cells decreased in each group, although they were mostly found in the vessel walls, and no significant difference was found between the groups (*p* = 0.253). Changes in cellular proliferation were detected according to Ki-67 staining levels at the wound edge. We found more staining cells in NTAPG compared to others on days 7 and 14 after injury, and this difference was significant (*p* < 0.001). On day 21, the expression levels of Ki-67 in the mucosal area decreased in all groups (Figure 5 and Figure 6; Table S3).

**Figure 7:**
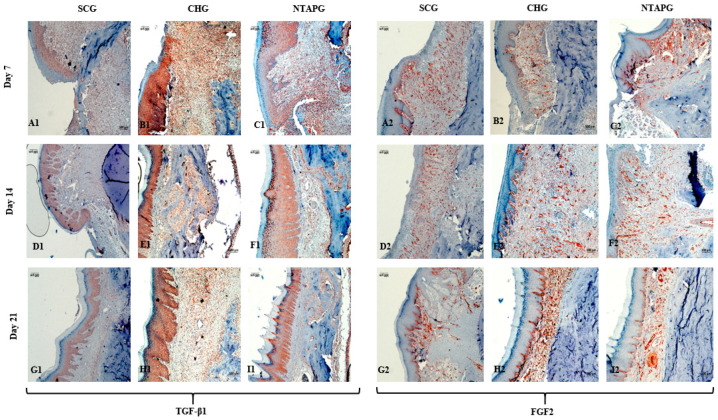
Immunohistochemical staining of TGF-β1 and FGF2 in the SCG, CHG and NTAPG at post-wounding days 7, 14, and 21. Panels **A1**–**C1**, **D1**–**F1**, and **G1**–**I1** represent TGF-β1 staining in SCG, CHG, and NTAPG on days 7, 14, and 21, respectively. Panels **A2**–**C2**, **D2**–**F2**, and **G2**–**I2** represent FGF2 staining in the same groups and corresponding time points. TGF-β1 expression was consistently significantly higher in the NTAPG compared to the other groups (*p* < 0.05) (**A1**–**I1**), and decreased in all groups by day 21 (**G1**–**I1**). The lowest FGF2 levels were observed in the SCG, whereas the highest levels were detected in the NTAPG on days 7 and 21 post-wounding (*p* < 0.05) (**A2**,**C2**,**G2**,**I2**). The magnification was ×100 in all the panels; the scale bar was 100 μm.

The peak level of TGF-β1 was found on the 7th day after injury and then the expression level decreased until day 21 post-wounding in both groups in the mucosal and submucosal area. The level of TGF- β1 expression was always significantly higher in the NTAPG than in the CHG and SCG (*p* = 0.002 and *p* < 0.001). The TGF- β1 level of the three groups decreased on the 21st day, and there were significant differences among the three groups (*p* < 0.001). The measurements of FGF2-stained cell levels indicated that the lowest FGF2 levels were obtained in the SCG and highest FGF2 levels were obtained in the NTAPG on the 14th and 21st day post-wounding (*p* ˂ 0.001 and *p* = 0.002). For the 14th day measurement, all groups exhibited low FGF2 levels compared to the other days, and expression was always significantly higher in the NTAPG (*p* = 0.003, *p* ˂ 0.001 and *p* = 0.002) (Figure 5 and Figure 7; Table S3).

**Figure 8:**
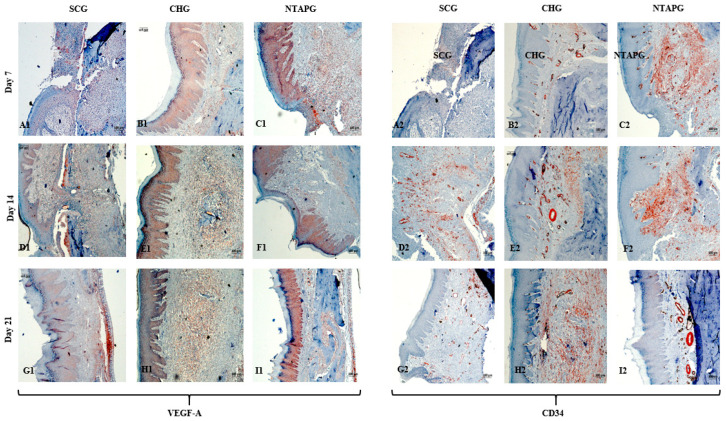
Immunohistochemical staining of VEGF-A and CD34 in the SCG, CHG and NTAPG at post-wounding days 7, 14, and 21. Panels **A1**–**C1**, **D1**–**F1**, and **G1**–**I1** represent VEGF-A staining in SCG, CHG, and NTAPG on days 7, 14, and 21, respectively. Panels **A2**–**C2**, **D2**–**F2**, and **G2**–**I2** represent CD34 staining in the same groups and corresponding time points. VEGF-A expression was significantly higher in the NTAPG on days 7 and 14 post-wounding (*p* < 0.05) (**C1**–**F1**). CD34 expression was significantly higher in the NTAPG on days 7, 14, and 21 (*p* < 0.05) (**C2**,**F2**,**I2**). The magnification was ×100 in all the panels; the scale bar was 100 μm.

The lowest VEGF-A levels were obtained in the SCG, and the highest levels were seen in the NTAPG in the mucosal area. At days 7 and 14 after injury, significantly higher levels of VEGF-A were detected in the NTAPG in comparison with the other groups (*p* < 0.001). For the 21st day measurement, the NTAP-treated and the CHG exhibited significantly higher VEGF-A levels compared to the SCG and CHG (*p* < 0.001). The results showed the highest CD34 expression for the NTAPG in all time measurements especially vascular area and submucosal area. On days 7 and 21 post-wounding, a significantly higher CD34 level was observed in the NTAPG compared with both the CHG and SCG (*p* = 0.005 and *p* = 0.001) and on day 14 significantly higher in NTAPG when compared with the CHG (*p* < 0.001) (Figure 5 and Figure 8; Table S3).

## 4. Discussion

Recently, non-thermal atmospheric plasma applications have been gradually developed and have been considered as a potential tool for wound management due to their beneficial effects on wound healing. Although plasma treatment has been used to promote wound healing in several studies, the mechanisms of this therapy remain not fully identified [10]. Therefore, we aimed to investigate the effect of NTAP on wound healing both clinically and on a cellular basis. The results of this present study revealed that NTAP-treatment had potential to promote oral-wound healing in rats’ palatal mucosa, used as a model. In histological sections, the NTAPG showed increased fibroblast proliferation, collagen deposition, vascularization, and accelerated re-epithelialization, and also, the inflammatory cells were fewer in the NTAPG than in other groups.

In terms of immunohistochemistry parameters, our study revealed that NTAP treatment increased TGFβ1, VEGF-A, FGF2, and CD34 levels in sections. It is well-reported that endogenous growth factors released from different cells coordinate the complex wound-healing process [19]. Additionally, they control the function, migration, differentiation, or survival of cells, as well as the organization of cell division and growth [20]. NTAP has been stated to briefly change the local cellular microenvironment, promoting the release of growth factors and signaling via the activation of intracellular pathways and modulation of initial inflammatory responses, thereby enhancing tissue regeneration and wound healing [4,11]. TGF-β is a super family of proteins that are released by fibroblasts, platelets, and macrophages, and have a crucial role in all phases of wound healing. TGF-β promotes angiogenesis, granulation tissue formation, collagen deposition and soft tissue remodeling [21]. TGF-β also stimulates fibroblasts to proliferate and differentiate into myofibroblasts, which control wound contraction [22]. In the wound healing process, re-epithelialization is very important, as it is necessary for cell migration. During wound repair, keratinocyte functions are regulated by TGF-β, which stimulates keratinocytes to express integrin, which eases the migratory component of re-epithelialization [23]. In an in vivo and in vitro study, Arndt et al. showed that TGF-ß1 and TGF-ß2 were induced in keratinocytes as well as in fibroblasts after the NTAP treatment [11]. The results of gene expression analysis showed that NTAP exposure of gingival fibroblasts resulted in increased mRNA expression of TGF-β1 compared to the control [24]. Choi et al. reported that the expression of TGF-β1 was increased in mouse skin after treatment with NTAP for two weeks [4]. While the present study specifically evaluated TGF-β1 expression, several studies indicate that other TGF-β isoforms, particularly TGF-β2 and TGF-β3, may also participate in NTAP-mediated tissue repair, possibly through distinct regulatory mechanisms in epithelial and mesenchymal compartments. Consistent with previous findings, our results demonstrated a significant increase in TGF-β1 levels in NTAP-treated sections compared with untreated sections, supporting the potential role of NTAP in modulating key profibrotic and regenerative signaling pathways during oral wound healing.

Another crucial growth factor in the wound-healing process is FGF2. In addition, FGF2 is chemotactic for several cell types such as endothelial cells and macrophages and also stimulates the proliferation and migration of fibroblasts [25]. Our results revealed that NTAP treatment significantly increased FGF2 expression compared with other groups. Several studies also suggested a specific role for NTAP in FGF2 expression. In one study, Cui et al. reported that the secretion of FGF2 increased in plasma-treated human keratinocytes without associated cell toxicity. In this study, the mRNA expression of angiogenic growth factors had been measured by real-time PCR, and FGF2 mRNA levels were significantly increased 24 h after exposure to plasma for 30 s, compared to those in untreated controls (*p* ˂ 0.05) [26]. Similarly, Kalghatgi et al. reported that low-dose plasma application induced proliferation of endothelial cells through reactive-oxygen-species-enhanced FGF2 release [27]. Moreover, NTAP was found to stimulate FGF2 in vitro to produce human gingival fibroblasts at 72 h after irradiation [28]. Arjunan et al. analyzed the effects of plasma irradiation on FGF2 production and reported that this cellular process was significantly increased by irradiation [29]. These studies support our results for FGF2, and both studies suggest a favorable effect of plasma irradiation on tissue wound healing.

Angiogenesis is the physiological basis of wound healing, which is organized by cytokines and growth factors via several signaling pathways that support or inhibit wound healing. Various growth factors are expressed and affect regulation during angiogenesis, but VEGF (a growth factor specific to vascular endothelium) is known to be quite important in this process [30]. We used VEGF and CD34 immunochemical staining to evaluate the newly formed vessels in this study. VEGF has already been shown to influence wound healing in previous studies and the results of these studies showed that VEGF advanced the synthesis of the extracellular matrix, improved re-epithelialization, and increased angiogenesis during tissue repair [31]. We found that the wounds in the NTAP treatment groups had significantly increased levels of VEGF-A compared to the control and chlorhexidine groups (*p* < 0.001). Consistent with our study, Choi et al. showed that treatment with cold plasma induced the expression of VEGF genes in skin cells [32]. CD34 is another marker of tissue vascularization and represents the micro vessel density; therefore, it is highly expressed on the surface of regenerating endothelial cells [33]. Our results revealed an increase of CD34 levels in wounds treated with NTAP compared to non-treated wounds. The increase in these markers, which are indicative of vascularization on the wound surface, may support the view that plasma has a positive effect on vascularization during wound healing.

It is well-known that cell proliferation is one of the most important events in the wound-healing process. In our study, cell proliferation was evaluated with Ki67 (nuclear protein that is associated with cellular proliferation) and the presence of myofibroblasts with α-SMA immunohistochemical staining. α-SMA indicates the number of myofibroblasts or fibroblasts with smooth muscle contraction, which has a significant role in the healing process [34]. The findings of the present study revealed that the expression of Ki67 and α-SMA in the NTAPG was higher than in the chlorhexidine and control groups on day 14 after treatment, which indicates the NTAP mechanism of action. Similarly, Kleineidam et al. investigated the effect of NTAP on the human periodontal ligament (PDL) cells’ regenerative capacity, and they showed that NTAP treatment induced the expression of the proliferation marker Ki67 [35]. Delben et al. showed an upregulation of Ki67 on oral keratinocytes with treatment of CAP in an in vivo and in vitro model [36]. These findings showed that NTAP treatment promoted the wound-healing process by increasing cell proliferation, which in turn resulted in improved tissue regeneration. And also, it could be considered that increased proliferation of epithelium was caused by connective tissue activity, especially myofibroblast action. In this study, NTAP treated wounds showed significantly higher reductions in the wound area, which is suggestive of an increased myofibroblast activity and increased epithelial cell growth with NTAP treatment in oral tissues.

Re-epithelization is an important stage in wound healing, which is regulated by the complex action of various cytokines, mediators and growth factors. In this study, the re-epithelialization of the palatal wounds was evaluated with the measurement of the distances between the epithelial margins in each H&E section and the calculation of the wound-healing area in palatal specimens’ photographs. According to our findings, the wound width in the NTAPG was significantly smaller than in the chlorhexidine and control groups, on days 7 and 14 (*p* ˂ 0.001). And the epithelial thickness in the control group had lower dimensions than the NTAP-treated groups. Consistent with these results, previous studies have reported beneficial effects of cold plasma on wound healing by promoting re-epithelialization [37]. Vandersee et al. found that wounds treated with plasma, showed an earlier starting of the proliferative phase, thereby resulting in accelerated wound healing [38]. In another study, it was reported that plasma application provided improvement and 30% of reduction in mice wound area after 9 days of treatment [14].

According to our results in H&E-stained sections, the NTAPG showed increased vascularization, more regular collagen deposition, more fibroblast proliferation, and fewer inflammatory cells. Previous findings revealed that NTAP treatment increases fibroblast migration, resulting in the rapid closure of gaps in the wound [39]. In our study, the enhancement in the deposition of collagen fibers was likely caused by the activation of fibroblasts after NTAP treatment. Furthermore, the application of NTAP on palatal wounds not only caused the increase in collagen fibers but also enhanced the expression of various growth factors (FGF2, TGF-β, and VEGF-A); and the wound-healing-promoting results observed in our study may also be caused by these growth factors. Therefore, it can be concluded that growth factors expressed from the wound after NTAP treatment promoted the fibroblasts to produce and secrete several types of extracellular matrix components and thus caused the accelerated wound-healing process. In the present study, the NTAPG showed significantly higher reduction in the wound area, which is suggestive of an increased myofibroblast activity and resulted in an increase in epithelial cell growth.

In the oral cavity, the healing of secondary wounds is at high risk of developing bacterial infection due to large commensal oral flora and this can impair the normal sequence of the healing process. Furthermore, NTAP is being currently investigated as a wound antiseptic and several studies have revealed the antibacterial effect both in vivo and in vitro [40,41]. These findings support the primary antiseptic effect of NTAP and in this way NTAP may enhance wound healing in infected or contaminated wounds. In our study, there were no data to evaluate the presence of bacterial changes in any wounds so previous microbiological studies could not be supported. This can be considered as a limitation of this study. However, it is possible to hypothesize that the positive effects observed in our findings may be related to the antimicrobial efficacy of NTAP. Future investigations are required to address these limitations.

Along with the histological and immunohistochemical results, the operational features of the NTAP device used in this study match those reported in plasma medicine literature. The electro-optical calibration tests (I–V behavior and optical emission profile) showed stable argon plasma generation with spectral characteristics similar to earlier biomedical NTAP studies [5,11]. This indicates that the plasma-tissue interactions in our model occurred under standard, well-understood plasma chemistry conditions. Additionally, our prior research on NTAP in experimental periodontitis models supports the reproducibility of the plasma application protocol used here, both in device settings and biological responses [15]. Collectively, these findings reinforce the methodological foundation of the current study and align it with established NTAP research.

## 5. Conclusions

This study was designed to examine molecular mechanisms and basic components that affect wound healing using NTAP. In conclusion, within the limitations of the present study, it was found that NTAP enhanced oral wound healing in rats’ palate with high cell proliferation and accelerated wound closure. In addition, re-epithelization, fibroblast proliferation, angiogenesis, and collagen deposition were increased with NTAP treatment. Furthermore, NTAP promoted the expression of growth factors, such as VEGF-A, FGF2, and TGF-β involved in the enhancement of angiogenesis and re-epithelization. In line with all these results, NTAP can be considered as a beneficial tool for oral wound management. Furthermore, additional studies are required to clarify the beneficial effects of NTAP on oral-wound healing in humans and with respect to standardization treatment modalities. A limitation of the present study is the absence of direct evaluation of pro-inflammatory markers; therefore, although NTAP demonstrated clear histological and immunohistochemical features consistent with reduced inflammation, its specific effects on pro-inflammatory signaling pathways could not be directly assessed and should be addressed in future studies.

## Data Availability

Research data are not shared.

## Supplementary Materials

The following supporting information can be downloaded at https://www.mdpi.com/article/10.3390/biomedicines14010089/s1, Table S1: Wound surface areas and the distance between the epithelial margins in all groups at each time points after wounding; Table S2: Collagen, vascularization, and inflammation scores in H&E sections of all groups at each time points after wounding; Table S3: Immunohistochemical stained for α-SMA, Ki67, TGF-β, FGF2, VEGF-A and CD34 scores in all groups at each time points after wounding.

## Abbreviations

α-SMA	Alpha-smooth muscle actin
ANOVA	Analysis of variance
CAP	Cold atmospheric plasma
CD34	Cluster of differentiation 34
CHG	Chlorhexidine gluconate group
DAB	3,3′-Diaminobenzidine
EBM	Extracellular basement membrane
ECM	Extracellular matrix
EDTA	Ethylenediaminetetraacetic acid
FGF-2	Fibroblast growth factor 2
H&E	Hematoxylin and eosin
HRP	Horseradish peroxidase
ILK	Integrin-linked kinase
IHC	Immunohistochemistry
Ki67	Proliferation marker Ki-67
NTAP	Non-thermal atmospheric plasma
NTAPG	Non-thermal atmospheric plasma-treated group
OD	Optical density
PBS	Phosphate-buffered saline
PDL	Periodontal ligament
ROS/RNS	Reactive oxygen species/reactive nitrogen species
SCG	Saline control group
TGF-β	Transforming growth factor beta
VEGF-A	Vascular endothelial growth factor A
