# Supplementary material for: Nonthermal Atmospheric Plasma Modulates Palatal Wound Healing in Rats: A Morphometric, Histopathologic and Immunohistochemical Analysis
Source: Biomedicines. 2026 Jan 1;14(1):89. doi: 10.3390/biomedicines14010089 (PMC12839261; doi:10.3390/biomedicines14010089)
Supplement: Supplementary file 1 [file biomedicines-14-00089-s001.zip › biomedicines-4034220-supplementary.pdf]

Table S1. Wound surface areas and the distance between the epithelial margins in all groups at each time points after wounding.

	Wound areas (mm^2) (means ± SD)			The distance between the epithelial margins (μm) (means ± SD)		
Days post-wounding	Saline Control	Chlorhexidine gluconate	NTAP-treated	Saline Control	Chlorhexidine gluconate	NTAP-treated
7th	10.98 ± 1.18*†‡	7.24 ± 1.14*†	6.03 ± 0.89*‡	1753.50 ± 91.37*†‡	1199,50 ± 63.98*†§	926.66 ± 66.99*‡§
14th	7.24 ± 0.94*†‡	4.78 ± 1.0*†§	1.64 ± 0.95*‡§	1054.83 ± 44.07*†‡	395.50 ± 43.09*†§	178.33 ± 54.36*‡§

ANOVA and post-hoc test (Scheffe). P <0.001 in all groups. Statistically significant at P <0.05; * Compression in both groups at the same time point; †SCG compared with CHG at the same time point; ‡NTAPG compared with SCG at the same time point; §NTAP compared with CHG at the same time point.

Table S2. Collagen, vascularization, and inflammation scores in H&E sections of all groups at each time points after wounding.

Scores	Days post-wounding	Saline Control (means ± SD)	Chlorhexidine gluconate (means ± SD)	NTAP-treated (means ± SD)	P
Collagen	7th	1 ± 0	1,3 ± 0,5	1 ± 0	0.119
	14th	1,5 ± 0,5	2,2 ± 0,4	2,5 ± 0,8	0.052
	21st	1,1 ± 0,8*‡	1,2 ± 0,4*	1,8 ± 0,5*‡	0.036
Vascularization	7th	1 ± 0*‡	1,3 ± 0,5*	2 ± 0*‡	0.002
	14th	1,2 ± 0,4*†‡	2 ± 0*†	2,3 ± 0,5*‡	0.001
	21st	1,2 ± 0,4	1 ± 0	1 ± 0	0.36
Inflammation	7th	2,7 ± 0,5*‡	2 ± 0*	2 ± 0*‡	0.001
	14th	2 ± 0,6*‡	2,3 ± 0,5*	1,3 ± 0,5*‡	0.043
	21st	1,5 ± 1*‡	1 ± 0*	0,8 ± 0,4*‡	0.015

Kruskal Wallis and Dunn's test. Statistically significant at P <0.05; * Compression in both groups at the same time point; †SCG compared with CHG at the same time point; ‡NTAPG compared with SCG at the same time point; §NTAP compared with CHG at the same time point.

Table S3. Immunohistochemical stained for α-SMA, Ki67, TGF-β, FGF2, VEGF-A and CD34 scores in all groups at each time points after wounding.

	Days post-wounding	OD			
		Saline Control	Chlorhexidine gluconate	NTAP-treated	P
α-SMA	7th	0.077 ± 0.018*†	0.09 ± 0.008*†	0.068 ± 0.018*	0.046
	14th	0.010 ± 0.01*‡	0.081 ± 0.01*§	0.091 ± 0.008*‡§	0.013
	21st	0.053 ± 0.016	0.037 ± 0.012	0.022 ± 0.012	0.253
Ki67	7th	0.094 ± 0.006*†‡	0.129 ± 0.009*†§	0.071 ± 0.009*‡§	<0.001
	14th	0.154 ± 0.014*†	0.131 ± 0.019*†§	0.098 ± 0.008*§	<0.001
	21st	0.103 ± 0.011*	0.124 ± 0.016*§	0.082 ± 0.009*§	0.001
TGF-β1	7th	0.088 ± 0.009*‡	0.207 ± 0.057*§	0.087 ± 0.011*‡§	0.002
	14th	0.068 ± 0.010*‡	0.087 ± 0.010*	0.088 ± 0.014*‡	0.002
	21st	0.067 ± 0.010*‡	0.114 ± 0.018*§	0.063 ± 0.009*‡§	<0.001
FGF2	7th	0.077 ± 0.018*‡	0.090 ± 0.008*	0.068 ± 0.012*‡	0.003
	14th	0.108 ± 0.010*†‡	0.081 ± 0.010*†§	0.091 ± 0.008*‡§	<0.001
	21st	0.053 ± 0.016*‡	0.037 ± 0.012*§	0.022± 0.012*‡§	0.002
VEGF-A	7th	0.083 ± 0.002*†‡	0.053 ± 0.003*†§	0.075 ± 0.002*‡§	<0.001
	14th	0.091 ± 0.003*†‡	0.124 ± 0.003*†§	0.064 ± 0.002*‡§	<0.001
	21st	0.066± 0.002*†‡	0.126 ± 0.003*†§	0.052 ± 0.009*‡§	<0.001
CD34	7th	0.035 ± 0.010*‡	0.032 ± 0.003*§	0.086 ± 0.007*‡§	0.005
	14th	0.032 ± 0.018*†	0.081 ± 0.014*†§	0.072 ± 0.019*§	<0.001
	21st	0.064 ± 0.011*‡	0.091 ± 0.011*§	0.031 ± 0.008*‡§	0.001

For non-normally distributed datas; Kruskal Wallis and post-hoc test (Dunn). For normally distributed datas; ANOVA and post-hoc test (Scheffe) Statistically significant at $P < 0.05$; * Compression in both groups at the same time point; †SCG compared with CHG at the same time point; ‡NTAPG compared with SCG at the same time point; §NTAPG compared with CHG at the same time point. OD: Optical Density.
